# Quantify the role of superspreaders -opinion leaders- on COVID-19 information propagation in the Chinese Sina-microblog

**DOI:** 10.1371/journal.pone.0234023

**Published:** 2020-06-08

**Authors:** Fulian Yin, Xinyu Xia, Nan Song, Lingyao Zhu, Jianhong Wu

**Affiliations:** 1 College of Information and Communication Engineering, Communication University of China, Beijing, PR China; 2 Fields-CQAM Laboratory of Mathematics for Public Health, Laboratory for Industrial and Applied Mathematics, York University, Toronto, Canada; Centre National de la Recherche Scientifique, FRANCE

## Abstract

**Backgroud:**

Effective communication of accurate information through social media constitutes an important component of public health interventions in modern time, when traditional public health approaches such as contact tracing, quarantine and isolation are among the few options for the containing the disease spread in the population. The success of control of COVID-19 outbreak started from Wuhan, the capital city of Hubei Province of China relies heavily on the resilience of residents to follow public health interventions which induce substantial interruption of social-economic activities, and evidence shows that opinion leaders have been playing significant roles in the propagation of epidemic information and public health policy and implementations.

**Methods:**

We design a mathematical model to quantify the roles of information superspreaders in single specific information which outbreaks rapidly and usually has a short duration period, and to examine the information propagation dynamics in the Chinese Sina-microblog. Our opinion-leader susceptible-forwarding-immune (OL-SFI) model is formulated to track the temporal evolution of forwarding quantities generated by opinion leaders and normal users.

**Results:**

Data fitting from the real data of COVID-19 obtained from Chinese Sina-microblog can identify the different contact rates and forwarding probabilities (and hence calculate the basic information forwarding reproduction number of superspreaders), and can be used to evaluate the roles of opinion leaders in different stages of the information propagation and the outbreak unfolding.

**Conclusions:**

The parameterized model can be used to nearcast the information propagation trend, and the model-based sensitivity analysis can help to explore important factors for the roles of opinion leaders.

## Introduction

In the absence of effective treatment and vaccine, the control of the novel coronavirus, COVID-19, in China was achieved by an unprecedented massive non-medical public health interventions involving gradually improved rapid diagnostics, contact tracing followed by quarantine and isolation. These interventions induced significant interruption of social-economic activities, and therefore their successful implementation by a large population clearly indicated the effective propagation of public health information through social media. Evidence shows the substantial role played by the social media superspreaders—the opinion leaders to get the message out to the community. For example, on January 20^th^ of 2020, Nanshan Zhong, an academician of the Chinese Academy of Engineering, personally confirmed the occurrence of the human-to-human transmission of the COVID-19 [1]. His confirmation, forwarded by opinion leaders and followed by their fans, changed the perception of the public about the risk of this emerging infectious disease in a very short period time right before the traditional Chinese New Year. A lesson learnt in this painful Chinese experience is the early and timely warning of a public health risk, and an experience gained from initial success of containing the disease is the resilience of the citizens which is informed by accurate public health messaging. It is important to examine how opinion leaders have influenced public opinion and information propagation in social media in order to design effective communication strategies for the on-going battle against COVID-19 and future pandemic outbreaks.

Our focus here is to develop an appropriate model framework that can be used to analyze the influence of opinion leaders, in different stages of single relevant information propagation with short duration periods, during a public health emergency event like COVID-19. This kind of model framework incorporating the involvement of opinion leaders in different stages of an event is lacking in the literature to our best knowledge. Considering there exists susceptible users, opinion leaders and normal forwarding users in social media and these kinds of users may impact public opinion, here we propose an opinion-leader susceptible-forwarding-immune (OL-SFI) dynamics model based on the forwarding quantity generated by opinion leaders and normal users in different stages of a hot topic dissemination through online social media during the COVID-19 outbreak in China. We address this stage-specific involvement of opinion leaders by considering the different contact rates and forwarding probabilities with different stages and by analyzing the propagation mechanism with the participation of opinion leaders at different times.

There have been intensive studies on opinion leaders, focusing on opinion leaders’ identification [2–4], opinion leaders’ characteristics [5, 6] and the role of opinion leaders [7, 8]. We found limited studies on the influence of opinion leaders on information propagation, which is important for the dissemination of public opinions to impact public health policy implementation during an emergency situation.

In the field of information dynamics, specially the propagation of rumor, infectious disease epidemiological models have been used, these include for example the susceptible-infected (SI) model [9, 10], the susceptible-exposed-infected-recovered (SEIR) model [11, 12], the susceptible-infected-recovered (SIR) model [13, 14] and the susceptible-infected-susceptible (SIS) [15] model. In these formulations, information propagation was analyzed by stratifying users into three classes: heard rumor (ignorants), actively spreading rumor (spreaders) and no longer spreading rumor (stiflers) [16] in mass media platforms.

With the development of network technology, several researchers introduced important factors into basic models in order to make the simulation process more closely fit the actual situations. In 2015, Zhang et al [17] studied the cumulative effects of memory on rumor spreading by using the data set of Chinese Sina-Microblog, and proposed a rumor spreading model which examined how the memory affected rate changes over time in artificial network and a real social network. Zhang et al [18] developed the dynamic 8-state ignorance-carrier-spreader-advocate-removal (ICSAR) rumor propagation model to study the mechanism of rumor propagation and then analyzed eight influencing factors including information attraction, objective identification of rumors, subjective identification of people, the degree of trust of information media, spread probability, reinforcement coefficient, block value and expert effects. Wang et al [19] proposed a novel susceptible-infected-removed (SIR) model by introducing the trust mechanism and investigated the critical threshold and the final size of the rumor spreading, which greatly reduced the maximum rumor influence. Liu et al [20] introduced a modified rumor spreading model called SIRE, which compared to the traditional rumor spreading model, had included the stifler’s broadcasting effect and social intimacy degree between people. Hu et al [21] made an assumption that there were three attitudes towards rumors among people, based on that assumption, then established a susceptible-hesitating-affected-resistant (SHAR) model, which considered individuals’ different attitudes towards rumor spreading. Su et al [22] proposed an improved model entitled microblog-susceptible-infected-removed (Mb-SIR) for information propagation by explicitly considering the user’s incomplete reading behavior, and tested the effectiveness of the model using real data from Sina-microblog, which was more accurate in describing the information propagation in the microblog. Cheng et al [23] established a stochastic epidemic model considering that individual whether or not was infected by the neighbor spreader greatly depended on the trustiness of ties between them.

However, studies mentioned above fail to take into account the great role of opinion leaders in information dissemination. In comparison, a few other studies incorporated “opinion leader” into traditional dynamics models. Liu et al [24] realized that messages were passed on from one user to another and numerous individuals were influenced by a relatively small portion of users, as also known as “super-spreaders” and then proposed a dynamics model to characterize the super-spreading phenomenon with considering the timeliness of the influence of super-spreaders in tweet information propagation. In their study, it was suggested that super-spreaders can produce a great impact when they first entered the event, however, they might stop being highly influential because they were not interested in this event. After a while, their interest in this event revived, so they rejoined it and then they turned into normal infectious users, who no longer had the same influence as before.

Traced back to a period when studying epidemic propagation was popular and scholars noticed networks about system of epidemiological relevance presented a heterogeneous topology [25]. Indeed, the existence of “super-spreaders” showing heterogeneity in epidemic had been investigated long ago. In 1980, in the area of epidemic, Kemper [26] studied SIR model and SIS model of epidemic for one population with superspreaders considering the constant degree by which superspreaders transmission rates exceeded “average” rates throughout the process. Then [27] noticed the core members of gonorrhea, who can also be called superspreaders, were few in number but had strong infectivity, so they set different parameters between core group and noncore group during the whole infectious period to reflect the otherness of two groups and two sets of parameters did not change with the process. These studies on superspreaders can effectively give strategies to control the epidemic when the state of dynamic system will not be changed by superspreaders.

However, different from effects superspreaders over epidemics in aforementioned pieces of literature, in social networks, with the participation of different opinion leaders at distinct times, the information propagation environment will change successively so that the state of dynamic system will be changed. Therefore, creating a model with piecewise dynamics is important and necessary. In this paper, we consider the average contact rate and forwarding probability of the propagation in a population at different stages when some opinion leaders enter the discussion of public health emergencies. By distinguishing between the contributions of opinion leaders and normal users to information propagation and analyzing the public opinion propagation mechanism with the participation of opinion leaders about COVID-19, we are able to quantify the influence of opinion leaders in information dissemination.

## Methods

**Fig 1:**
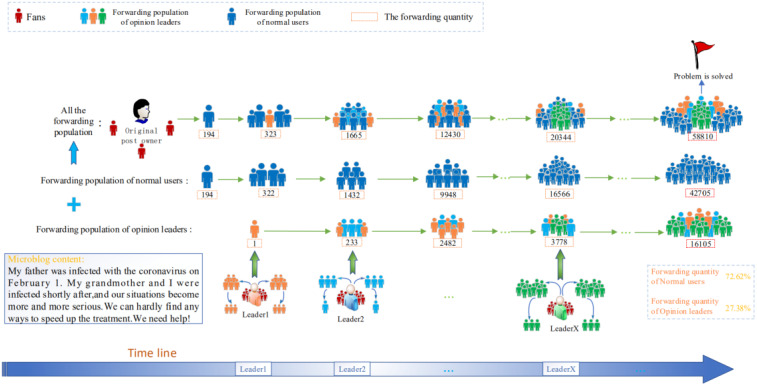
An illustration of a message receiving public attention after the involvement of several opinion leaders.

Fig 1 demonstrates the information propagation involving the involvement of several opinion leaders. A young girl in the epicenter, Wuhan/China, posted a message seeking for help to find ways to speed up the treatment for her family. At first, this Weibo was forwarded by some enthusiastic normal users shown in dark blue persons, but the forwarding quantity was very limited and increased slowly until some opinion leaders in the light blue/green/orange color who were followed by large crowds started to forward the Weibo leading to surging forwarding quantity and finally received attention by the public health agency and patients were hospitalized. Examples provided in subsequent sections contribute further illustrations with specific forwarding quantities.

Different from the spread of rumors and other traditional hot events, much information about the epidemic needs more attention, such as the help message shown in Fig 1. However, the exposure rate of normal users is limited if only a small fan groups involved, making their information difficult to arouse public concern. With the continuous development of the epidemic, there is a high level of demand that opinion leaders participate in spreading the information for getting more public attention. As the forwarding is an important way of information propagation, in this paper, we build the opinion-leader susceptible-forwarding-immune (OL-SFI) dynamics model with considering the distinction between the influence of opinion leaders and normal users.

**Fig 2:**
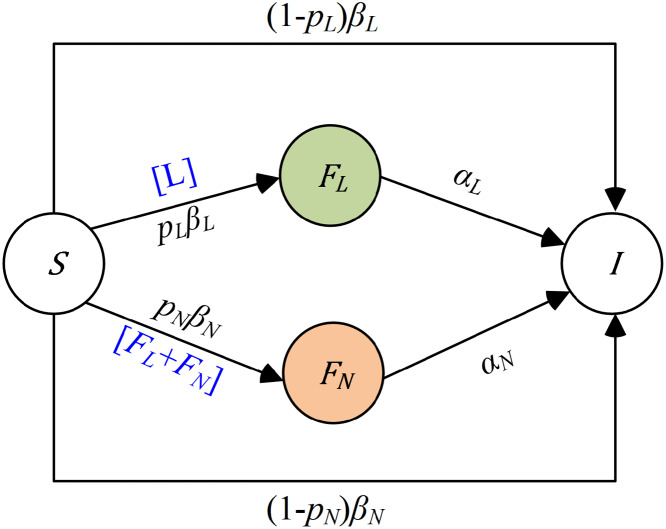
A schematic diagram to illustrate one information spreading in the population with four independent states: The susceptible state (*S*), the forwarding state influenced by opinion leaders (*F*_*L*_), the forwarding state influenced by normal users (*F*_*N*_) and the immune state (*I*). ‘[*L*]’ represents an opinion leader, as a superspreader, who has the ability to influence a susceptible user’s state after contact, ‘[*F*_*L*_ + *F*_*N*_]’ represents a normal user influenced by an opinion leader or a normal user, who has the ability to influence a susceptible user’s state after contact.

The propagation dynamics model based on the forwarding quantity of COVID-19 constructed in this paper is shown in Fig 2. Here we only pay attention to the accessible population in the process of one information propagation and consider the different influence caused by the forwarding of opinion leaders and normal users respectively. The experimental dataset includes three kinds of cumulative forwarding quantities at different times, the number of fans of superspreaders and different times for superspreaders to participate in the information. And our method of data collection accords with the terms and conditions of Chinese Sina-microblog API. We make an assumption that all users (*N*) remain unchanged which could be contacted in the course of information propagation are in a closed environment, and only focus on the forwarding behavior of individuals. Each individual user may be in one of four states at any given time: the susceptible state (*S*), the forwarding state influenced by opinion leaders (*F*_*L*_), the forwarding state influenced by normal users (*F*_*N*_) and the immune state (*I*).

With the participation of different opinion leaders, the communication environment will change successively. One important distinction between the traditional opinion leader dynamics models and our OL-SFI model is the piecewise characteristic of different parameters through the participation of opinion leaders. At the same time, our model emphasizes the role of opinion leaders by distinguishing the differences in the contact rates and forwarding probabilities of opinion leaders and normal users.

Considering the number of opinion leaders is less than normal users in the process of propagation about COVID-19 in Chinese Sina-microblog and the identification criterion of opinion leaders is that they have more than 1000 fans [28], the piecewise function of the number of opinion leaders at each stage can be expressed as:

$$\begin{array}{c} f\left(t\right)=\{\begin{array}{c} 0,0\leq t<t_{1}\\ m_{1},t_{1}\leq t<t_{2}\\ m_{2},t_{2}\leq t<t_{3}\\ \;\ldots \\ m_{n},t_{n}\leq t<t_{n+1} \end{array}, \end{array}$$ (1)

where *t* ∈ [0, *t*_1_] denotes the first stage without the participation of opinion leaders, *t* ∈ [*t*_1_, *t*_2_], *t* ∈ [*t*_2_, *t*_3_], …, *t* ∈ [*t*_*n*_, *t*_*n*+1_] denote the stages with different opinion leaders, and *m*_1_, *m*_2_, …, *m*_*n*_ represent the number of opinion leaders at each stage.

Correspondingly, at each stage, susceptible users can contact one information forwarded by an opinion leader with an average exposure rate *β*_*L*_(*t*) and forward it with the forwarding probability *p*_*L*_(*t*), or contact one information forwarded by a normal user with an average exposure rate *β*_*N*_(*t*) and forward it with the forwarding probability *p*_*N*_(*t*). These parameters are also in the form of piecewise functions:

$$\begin{array}{c} \beta_{L}\left(t\right)=\{\begin{array}{c} 0,\;0\leq t<t_{1}\\ \beta_{L1},t_{1}\leq t<t_{2}\\ \beta_{L2},t_{2}\leq t<t_{3}\\ \;\ldots \\ \beta_{Ln},t_{n}\leq t<t_{n+1} \end{array},p_{L}\left(t\right)=\{\begin{array}{c} 0,0\leq t<t_{1}\\ p_{L1},t_{1}\leq t<t_{2}\\ p_{L2},t_{2}\leq t<t_{3}\\ \;\ldots \\ p_{Ln},t_{n}\leq t<t_{n+1} \end{array}, \end{array}$$ (2)

$$\begin{array}{c} \beta_{N}\left(t\right)=\{\begin{array}{c} \beta_{N0},0\leq t<t_{1}\\ \beta_{N1},t_{1}\leq t<t_{2}\\ \beta_{N2},t_{2}\leq t<t_{3}\\ \;\ldots \\ \beta_{Nn},t_{n}\leq t<t_{n+1} \end{array},p_{N}\left(t\right)=\{\begin{array}{c} p_{N0},0\leq t<t_{1}\\ p_{N1},t_{1}\leq t<t_{2}\\ p_{N2},t_{2}\leq t<t_{3}\\ \;\ldots \\ p_{Nn},t_{n}\leq t<t_{n+1} \end{array}. \end{array}$$ (3)

In addition, forwarding users influenced by opinion leaders and normal users can become “immune users” who are inactive to the information when they out of the active forwarding period with the piecewise average inactive rate *α*_*L*_(*t*) and *α*_*N*_(*t*):

$$\begin{array}{c} \alpha_{L}\left(t\right)=\{\begin{array}{c} 0,0\leq t<t_{1}\\ \alpha_{L1},t_{1}\leq t<t_{2}\\ \alpha_{L2},t_{2}\leq t<t_{3}\\ \;\ldots \\ \alpha_{Ln},t_{n}\leq t<t_{n+1} \end{array},\alpha_{N}\left(t\right)=\{\begin{array}{c} \alpha_{N0},0\leq t<t_{1}\\ \alpha_{N1},t_{1}\leq t<t_{2}\\ \alpha_{N2},t_{2}\leq t<t_{3}\\ \;\ldots \\ \alpha_{Nn},t_{n}\leq t<t_{n+1} \end{array}. \end{array}$$ (4)

Even though the number of opinion leaders is rare, in order to ensure the preciseness and scientificity of the model, we simultaneously consider the contribution of opinion leaders themselves to forwarding quantity and their huge influence on the increase of forwarding quantity, and obviously, the latter is more important for information propagation. In this paper, we obtain the following OL-SFI dynamics model:

$$\begin{array}{c} S^{\prime }\left(t\right)=-\beta_{L}\left(t\right)S\left(t\right)f\left(t\right)-\beta_{N}\left(t\right)S\left(t\right)\left(F_{L}\left(t\right)+F_{N}\left(t\right)\right), \end{array}$$ (5)

$$\begin{array}{c} F_{L}^{\prime }\left(t\right)=p_{L}\left(t\right)\beta_{L}\left(t\right)S\left(t\right)f\left(t\right)-\alpha_{L}\left(t\right)F_{L}\left(t\right), \end{array}$$ (6)

$$\begin{array}{c} F_{N}^{\prime }\left(t\right)=p_{N}\left(t\right)\beta_{N}\left(t\right)S\left(t\right)\left(F_{L}\left(t\right)+F_{N}\left(t\right)\right)-\alpha_{N}\left(t\right)F_{N}\left(t\right), \end{array}$$ (7)

$$\begin{array}{c} \begin{array}{cc} I^{\prime }\left(t\right)= & \left(1-p_{L}\left(t\right)\right)\beta_{L}\left(t\right)S\left(t\right)f\left(t\right)+\left(1-p_{N}\left(t\right)\right)\beta_{N}\left(t\right)S\left(t\right)\left(F_{L}\left(t\right)+F_{N}\left(t\right)\right)\\ & +\alpha_{L}\left(t\right)F_{L}\left(t\right)+\alpha_{N}\left(t\right)F_{N}\left(t\right), \end{array} \end{array}$$ (8)

where ′ = *d*/*dt* is the derivative with respect to *t*. In the above dynamics model, we mainly focus on the impact of opinion leaders on forwarding quantity in the dynamic system. The main reason is: Opinion leaders, as a special group, belong to forwarding users and have a very small number, which themselves have a slight impact on cumulative forwarding quantity but have the special ability which can extremely prompt information propagation in a real sense to drive more people to participate in forwarding behavior and become forwarding users. Meanwhile, we do not consider the dynamic process in which the activity of opinion leaders attenuates into the immune state because opinion leaders have a longer influence period of to COVID-19 but the outbreak duration of information related to COVID-19 is usually shorter.

In the model, we distinguish between forwarding behaviors influenced by opinion leaders and forwarding behaviors influenced by normal users which can be interpreted as follow:

**Opinion leaders**: Since an active opinion leader who forward the information will contact an average number of *β*_*L*_(*t*)*N* users per time and the probability that a user who has contacted the information is a susceptible user is *S*(*t*)/*N*, among which *p*_*L*_(*t*)*β*_*L*_(*t*)*S*(*t*) will choose to forward the information and (1 − *p*_*L*_(*t*))*β*_*L*_(*t*)*S*(*t*) will be insensitive to the information so they will not forward it. Hence, the average number of new forwarding users influenced by opinion leaders and direct immune users are *p*_*L*_(*t*)*β*_*L*_(*t*)*S*(*t*)*f*(*t*) and (1 − *p*_*L*_(*t*))*β*_*L*_(*t*)*S*(*t*)*f*(*t*) respectively. After a while, the forwarding users will be out of an active forwarding period so that lose the ability to influence susceptible users, and the number of inactive immune users will be *α*_*L*_(*t*)*F*_*L*_(*t*) per time. **Normal users**: Similarly, the average number of new forwarding users influenced by normal users and direct immune users are *p*_*N*_(*t*)*β*_*N*_(*t*)*S*(*t*)(*F*_*L*_(*t*) + *F*_*N*_(*t*)) and (1 − *p*_*N*_(*t*))*β*_*N*_(*t*)*S*(*t*)(*F*_*L*_(*t*) + *F*_*N*_(*t*)) respectively. And after a while, the number of inactive immune users will be *α*_*N*_(*t*)*F*_*N*_(*t*) per time.

Considering the whole propagation influenced by opinion leaders and normal users, we pay attention to the cumulative number of their forwarding users. We have

$$\begin{array}{c} C_{L}\left(t\right)=\int_{0}^{t}p_{L}\beta_{L}S\left(t\right)f\left(t\right)dt, \end{array}$$ (9)

$$\begin{array}{c} C_{N}\left(t\right)=\int_{0}^{t}p_{N}\beta_{N}S\left(t\right)\left(F_{N}\left(t\right)+F_{L}\left(t\right)\right)dt, \end{array}$$ (10)

represent the cumulative forwarding population affected by opinion leaders and normal users, respectively.

In particular, we pay more attention to the total number of cumulative forwarding populations for the entire information expressed as:

$$\begin{array}{c} C\left(t\right)=C_{L}\left(t\right)+C_{N}\left(t\right)+f\left(t\right)\approx C_{L}\left(t\right)+C_{N}\left(t\right), \end{array}$$ (11)

because *f*(*t*) ≪ *C*_*L*_(*t*) + *C*_*N*_(*t*) and we focus on the final size of the summative cumulative forwarding population $C_{\infty }=\lim_{t\to \infty }C\left(t\right)$.

**Public opinion reproduction ratio ℜ_*o*_**: Since the number of opinion leaders in each period is different and their influence is different, we define the reproduction ℜ_*o*_(*t*) to describe the outbreak of public opinion at each time *t*. We use the calculation method of basic reproduction number on the spreading of the epidemic in [29], and rewrite our model as follows:

$$\begin{array}{c} \dot{x}=M\left(x\right)-V\left(x\right), \end{array}$$ (12)

where $\dot{x}={\left(F_{L}\left(t\right),F_{N}\left(t\right)\right)}^{T}$ and

$$\begin{array}{c} M\left(x\right)=\{\begin{array}{c} p_{L}\left(t\right)\beta_{L}\left(t\right)S\left(t\right)f\left(t\right)\\ p_{N}\left(t\right)\beta_{N}\left(t\right)S\left(t\right)\left(F_{L}\left(t\right)+F_{N}\left(t\right)\right) \end{array}, \end{array}$$ (13)

$$\begin{array}{c} V\left(x\right)=\{\begin{array}{c} \alpha_{L}\left(t\right)F_{L}\left(t\right)\\ \alpha_{N}\left(t\right)F_{N}\left(t\right) \end{array}, \end{array}$$ (14)

Different from the traditional methods, we set the no information propagation equilibrium at any time *t* as the beginning time we interested in. We obtain

$$\begin{array}{c} M\left(x\right)=[\begin{array}{cc} 0 & 0\\ p_{N}\left(t\right)\beta_{N}\left(t\right)S\left(t\right) & p_{N}\left(t\right)\beta_{N}\left(t\right)S\left(t\right) \end{array}], \end{array}$$ (15)

and

$$\begin{array}{c} V\left(x\right)=[\begin{array}{cc} \alpha_{L}\left(t\right) & 0\\ 0 & \alpha_{N}\left(t\right) \end{array}]. \end{array}$$ (16)

The roots of the characteristic equation can deduce the eigenvalues of the matrix *MV*^−1^:

$$\begin{array}{c} |\lambda E-MV^{-1}|=|\begin{array}{cc} \lambda & 0\\ -\frac{p_{N}\left(t\right)\beta_{N}\left(t\right)S\left(t\right)}{\alpha_{L}\left(t\right)} & \lambda -\frac{p_{N}\left(t\right)\beta_{N}\left(t\right)S\left(t\right)}{\alpha_{N}\left(t\right)} \end{array}|=0. \end{array}$$ (17)

Because ℜ_*o*_ is not zero, we deduce:

$$\begin{array}{c} {ℜ}_{o}=\lambda =\frac{p_{N}\left(t\right)\beta_{N}\left(t\right)S\left(t\right)}{\alpha_{N}\left(t\right)}. \end{array}$$ (18)

The ℜ_*o*_ denotes the number of forwarding-population generated by the information related to COVID-19 at time *t* influenced by different numbers of opinion leaders, which is determined by the average exposures rate *β*_*N*_(*t*), the forwarding probability *p*_*N*_(*t*), the average inactive rate *α*_*N*_(*t*) and the susceptible users *S*(*t*). At time *t*, ℜ_*o*_ < 1 represents that forwarding population rapidly decline so the information propagation can never break out; ℜ_*o*_ > 1 means that the forwarding population grows exponentially initially influenced by opinion leaders.

**Parameter estimation method**: To use our OL-SFI model to explore some distinctions of qualitative behaviors for prediction, we use the LS method to estimate the model parameters and the initial data of our OL-SFI model. The parameter vector can be set as Θ = (*α*_*L*_, *p*_*L*_, *β*_*L*_, *α*_*N*_, *p*_*N*_, *β*_*N*_, *S*_0_), and the corresponding numerical calculation based on the parameter vectors for *C*_*L*_(*t*) and *C*_*N*_(*t*) are denoted by $f_{C_{L}}\left(k,\Theta \right)$ and $f_{C_{N}}\left(k,\Theta \right)$, respectively. The LS error function

$$\begin{array}{c} LS=\sum_{k=0}^{T}{\left|f_{C_{L}}\left(k,\Theta \right)-C_{Lk}\right|}^{2}+\sum_{k=0}^{T}{\left|f_{C_{N}}\left(k,\Theta \right)-C_{Nk}\right|}^{2} \end{array}$$ (19)

is used in our calculation, where *C*_*Lk*_ and *C*_*Nk*_ denote the actual cumulative forwarding populations affected by opinion leaders and normal users, here, *k* = 0, 1, 2, … is the sampling time.

## Results

### Data description

The number of cumulative forwarding users and fans of users can be collected through the Chinese Sina-microblog’s Application Programming Interface (API). Tables 1 and 2 list part of the original dataset shown in Fig 1, for an actual event from 13:10 on February 8, 2020 to 6:30 on February 9, 2020, which lasts about 17 hours from the beginning of the event to the end. Table 1 gives the cumulative forwarding population (*C*_*L*_) influenced by ten opinion leaders respectively and the cumulative forwarding population (*C*_*N*_) influenced by normal users at each time. It can be intuitively seen that ten opinion leaders entered the information propagation at different points in time and especially five opinion leaders forwarded this information in an intensive time, then some opinion leaders forwarded successively. Especially, Table 1 shows the phenomenon that cumulative forwarding population of normal users is indirectly influenced by the participation of opinion leaders to some extent, that is, some users will forward the information of normal users after opinion leaders let more users know this event. And Table 2 shows the number of opinion leaders with more than 1000 fans which corresponds to the criterion of opinion leaders, meanwhile, shows the number of the final forwarding population of each opinion leader.

**Table 1:** Cumulative forwarding population at different points in time.

t(10min)	Time	*C*_*L*_										*C*_*N*_
		I	II	III	IV	V	VI	VII	VIII	IX	X	
0	13:10 February 8, 2020	0										28
1	13:20	0										49
……	……	……	……	……	……	……	……	……	……	……	……	……
7	14:20	1										322
8	14:30	81										499
9	14:40	123	23									809
10	14:50	148	45	40								1432
11	15:00	172	58	149								1972
12	15:10	194	67	354	11							2552
13	15:20	215	70	569	18							3317
14	15:30	232	76	689	26	64						4348
15	15:40	242	85	897	34	170						5391
……	……	……	……	……	……	……	……	……	……	……	……	……
19	16:20	300	105	1533	148	382	14					9948
20	16:30	324	107	1670	172	409	29					11109
……	……	……	……	……	……	……	……	……	……	……	……	……
24	17:10	606	120	1980	227	525	61	1				15008
25	17:20	640	121	2034	232	545	75	6				15814
……	……	……	……	……	……	……	……	……	……	……	……	……
34	18:50	843	133	2411	298	618	577	15	8			21661
35	19:00	861	140	2438	302	626	661	17	34			22226
……	……	……	……	……	……	……	……	……	……	……	……	……
42	20:10	942	161	2665	329	653	1328	21	273	31	97	26088
43	20:20	947	166	2705	336	657	1819	21	305	81	158	26837
……	……	……	……	……	……	……	……	……	……	……	……	……
104	06:30 February 9, 2020	1145	319	3567	721	690	8167	257	1090	955	548	42705

**Table 2:** The number of fans and forwarding population of each opinion leaders.

	t(10min)	The number of fans	The number of final forwarding population
Leader I	7	1905854	1145
Leader II	9	352392	319
Leader III	10	1744183	3567
Leader IV	12	78406	721
Leader V	14	2144029	690
Leader VI	19	1269890	8167
Leader VII	24	5741	257
Leader VIII	34	409299	1090
Leader XI	42	1561825	955
Leader X	42	817185	548

**Fig 3:**
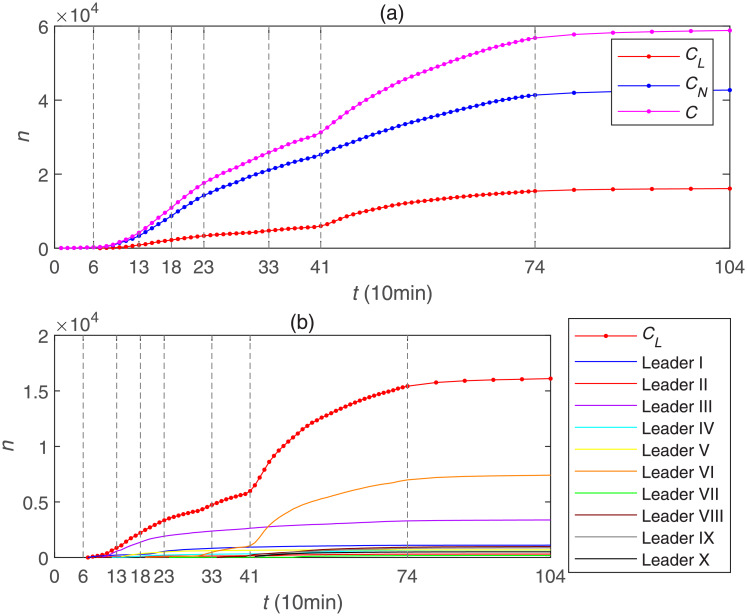
The cumulative number of forwarding population: (a) the cumulative forwarding population affected by opinion leaders, normal users and their sum; (b) the cumulative forwarding population affected by each opinion leader and their sum.

Fig 3(a) shows the trend of two kinds of cumulative forwarding population (*C*_*L*_) and (*C*_*N*_), and the summative cumulative forwarding population (*C*) combining (*C*_*L*_) and (*C*_*N*_) in Table 1. It can be explicitly found that when opinion leaders appeared, the number of summative cumulative forwarding population increased sharply compared to the previous uptrend. Due to the extensive and powerful influence of these opinion leaders, the number of summative cumulative forwarding population jumped from the initial small scale to the last big scale. Fig 3(b) shows the trend of the cumulative forwarding population influenced by each opinion leader and all opinion leaders in Table 1. According to the process of opinion leaders joining the dissemination of public opinion, we divide this information dissemination process into eight stages. The first stage is the non-opinion leader stage, that is, no opinion leader participates in the process of public opinion propagation from 0 minutes to 60 minutes. The second stage is the multiple opinion leaders joining stage. Since opinion leaders participate in the dissemination process intensively in this stage, in this paper, we will not analyze this participation stage in detail. In the third stage from 130 minutes to 180 minutes, a total of 5 opinion leaders joined the public opinion dissemination process. The next fourth stage, the fifth stage and the sixth stage are the single opinion leader joining stage, that is, only one opinion leader joins each stage. In the seventh stage from 410 minutes to 740 minutes, two opinion leaders join the public opinion dissemination process at the same time. Finally, the eighth stage is from 740 minutes to 1040 minutes, as the trend of this stage is stable so that we do not analyze it in this paper.

**Fig 4:**
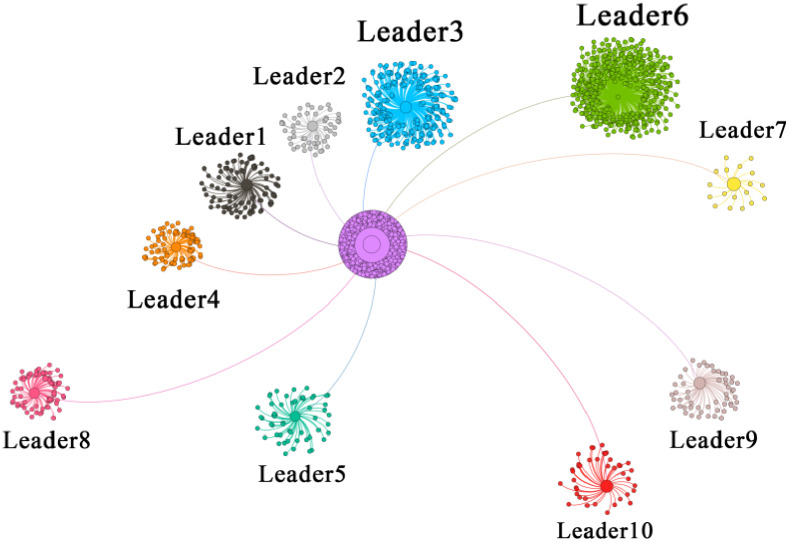
Forwarding network structure involving opinion leaders.

Fig 4 shows the structure of the forwarding network of this information posted by an original post owner and forwarded by normal users and opinion leaders. The length of lines between original post owner and opinion leaders represents the time when opinion leaders participate in the spread of the information, which means, the earlier they participate, the shorter the line will be, and this rule also applies between opinion leaders and normal forwarding users influenced by opinion leaders. Meanwhile, it can be seen that aggregative forwarding behavior is very common between opinion leaders and normal forwarding users influenced by opinion leaders so that several small circles with the same color of opinion leaders can be concentrated in this figure. It is obvious that opinion leaders have the ability to greatly affect the attitudes and behaviors of other users, resulting in thousands of users’ participation in the dissemination of public opinion. Opinion leaders greatly promote the outbreak of public opinion and bring this original information to the trend of widespread dissemination.

### Data fitting

**Fig 5:**
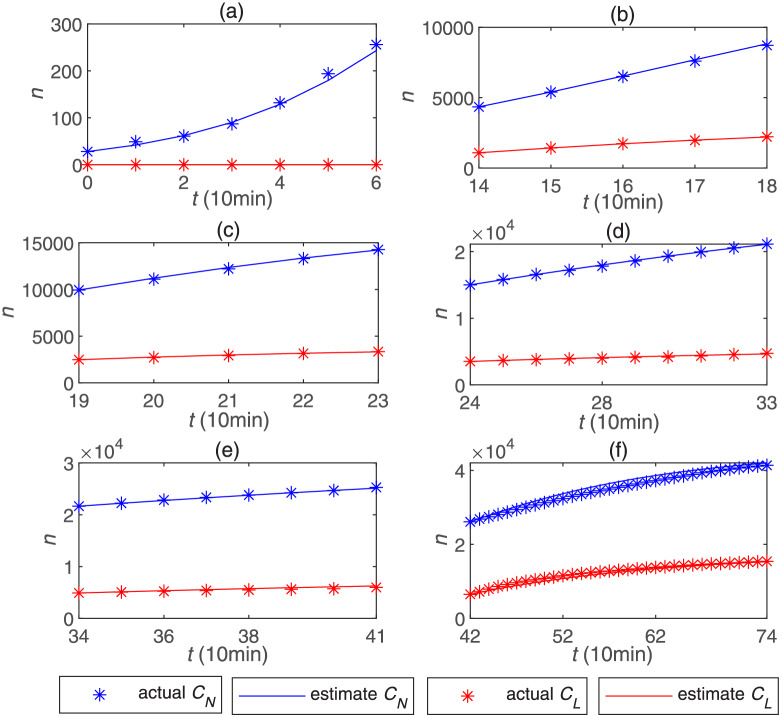
The data fitting results of COVID-19 in different stages: (a) Stage 1(0-60min); (b) Stage 3(140-180min); (c) Stage 4(190-230min); (d) Stage 5(240-330min); (e) Stage 6(340-410min); (f) Stage 7(420-740min).

As shown in Fig 5, we perform a piecewise data fitting on the real data in Table 1, where the blue star denotes the actual cumulative number of forwarding users affected by normal users, the red star denotes the actual cumulative number of forwarding users affected by opinion leaders, the blue line and the red line denote the estimated cumulative number of forwarding users affected by normal users and opinion leaders, respectively. Fig 5(a) is the first non-opinion leader stage, it is a traditional SFI [30] propagation process. From Fig 5(b) to 5(f), they are the stable opinion leader participation stages, and in each stage, our OL-SFI model all achieves accurate estimation.

Table 3 gives the parameter results of the data fitting at each stage. We can see that with the addition of opinion leaders, the initial value of the susceptible users *S_0_* is constantly increasing for their large number of fans, but the parameters *β_L_*,*β_N_*,*p_L_*,*p_N_*,*α_L_* and *α_N_* change irregularly. The average contact rate *β_L_* and *β_N_* are determined by the structure of the network, the forwarding probability *p_L_* and *p_N_* are affected by the users’ interest in this information and average rate *α_L_* and *α_N_* reflect the inactive character of users. With the participation of new opinion leaders, the normal users who forward opinion leaders’ information will affect their normal fans’ forwarding behavior later, hence, both instantaneous forwarding population *F_L_* influenced by opinion leaders and *F_N_* influenced by nomal users are changed at each stage.

**Table 3:** Parameter results.

t(10min)	f	*S_0_*	*α_L_*	*p_L_*	*β_L_*	*α_N_*	*p_N_*	*β_N_*
0-6	0	9.0412 × 10^3^	–	–	–	0.0208	0.0898	5.0676 × 10^−4^
14-18	5	5.2245 × 10^4^	0.0101	0.1497	0.0092	0.0095	0.2914	1.2045 × 10^−5^
19-23	6	5.7224 × 10^4^	0.0086	0.1572	0.0056	0.0109	0.1614	1.1377 × 10^−5^
24-33	7	1.2239 × 10^5^	0.0835	0.11	0.0017	0.0118	0.15	2.3450 × 10^−6^
34-41	8	1.4416 × 10^5^	0.0268	0.0632	0.0032	0.0089	0.142	1.0450 × 10^−6^
42-74	10	1.8229 × 10^5^	0.0935	0.133	0.0022	0.0116	0.21	7.2400 × 10^−7^

Table 2 shows the number of fans is not necessarily proportional to the final forwarding population of opinion leaders, which confirms the irregular feature mentioned above. The final forwarding population caused by an opinion leader with a larger number of fans may not be greater since the final forwarding population has a relationship with the vivacious level of fans, personal interest in the topic and is also related to the time when the opinion leaders join in. That is, the situation of communication is different, resulting in changes of parameters *β_L_*,*β_N_*,*p_L_*,*p_N_*,*α_L_* and *α_N_* at each stage, which validates the conclusions we have given above.

ℜ_*o*_ is the public opinion reproduction ratio, which is a function of time *t* as mentioned in section 3, representing the transmission capacity at each time. When a new opinion leader joins the public opinion propagation process, new susceptible users will be brought. At the same time, due to the participation of new susceptible users, the network structure changes, and individual characteristics in the network also change, resulting in changes in parameters such as the average exposure rate *β_L_* and *β_N_*. But overall, the addition of opinion leaders will cause an increase in the public opinion reproduction ratio ℜ_*o*_ as shown in Table 4, and promote the spread of public opinion.

**Table 4:** The results of the public opinion reproduction ratio ℜ_*o*_.

t(20min)	Opinion leader					
	0	I-V	I-VI	I-VII	I-VIII	I-X
0	19.7807					
1	19.4407					
……						
7	12.7138					
……						
13	2.7683					
14	1.9852	19.3027				
15	1.4141	17.1294				
……						
19	0.362	9.0549	9.6401			
20	0.2597	7.4501	8.0296			
……						
24	0.0725	3.1094	3.3928	3.6484		
25	0.0535	2.4612	2.6781	3.45		
……						
34	0.0045	0.286	0.2907	2.0049	2.4036	
35	0.0035	0.2257	0.2277	1.8817	2.2781	
……						
42	0.0007	0.045	0.0433	1.1993	1.5487	2.3893
43	0.0006	0.0359	0.0344	1.1242	1.464	2.2823
……						

## Discussion

**Fig 6:**
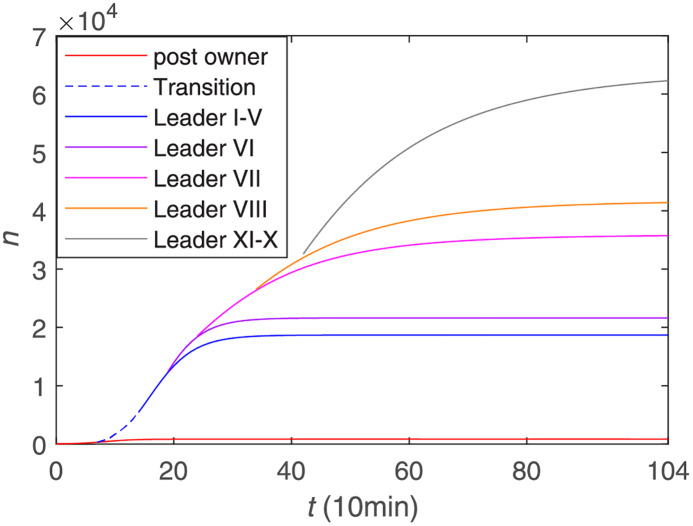
The cumulative number of forwarding users with the participation of opinion leaders.

We aimed to quantify the role of superspreaders -opinion leaders- on COVID-19 information propagation in the Chinese Sina-microblog. Fig 6 shows the trend of the cumulative forwarding population with the participation of opinion leaders, where the orange line denotes the cumulative forwarding population with the original post owner, and the remaining different color lines denote the different cumulative forwarding population after the participation of different opinion leaders’. From some perspectives, it can be seen that the increase in the cumulative forwarding population caused by different opinion leaders varies. Among them, opinion leader 7 has the greatest effect in promoting the development of public opinion, which is related to the reasons we analyzed above, such as the colossal number of fans, the interest in the topic and so on. As a whole, it can be seen that before the opinion leaders join, the public opinion outbreaks slowly. With the addition of the first five opinion leaders, the cumulative forwarding population begins to increase and public opinion breaks out. When the curve is nearly flat, the participation of new opinion leaders continues to increase the cumulative forwarding amount, that is, the addition of the opinion leaders gradually promote the dissemination.

**Fig 7:**
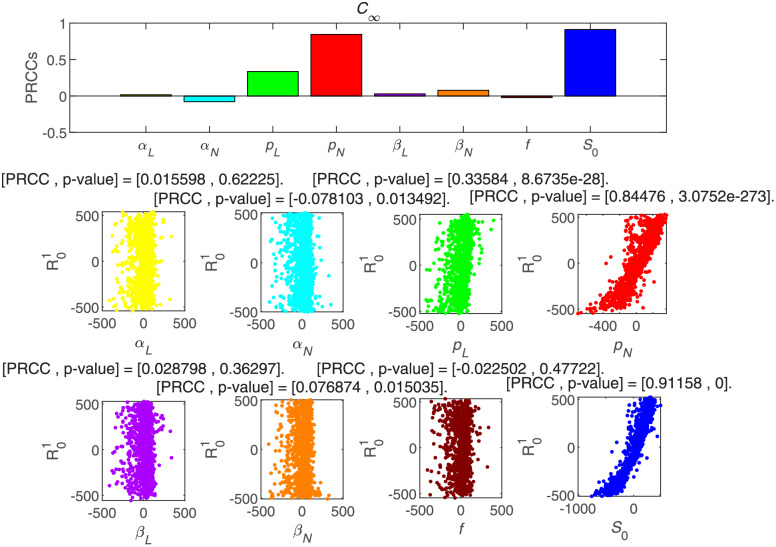
PRCC results and PRCC scatter plots with index *C*_∞_ of different parameters.

To further analyze the different parameters responsible for the OL-SFI model, we use the partial rank correlation coefficients (PRCCs) [31]based on 1000 samples for various input parameters against the threshold condition to evaluate the sensitivity. According to the histogram and scatter diagram of $R_{0}^{1}$ dependence, when the correlation is positive, it means that with the increase of the value of the parameter, the corresponding index value will increase. On the contrary, when the correlation is negative, the index will decrease as the parameter decreases. Fig 7 shows the final size of the cumulative forwarding population is strongly positively affected by forwarding probabilities *p_L_*, *p_N_* and the initial susceptible users *S_0_*. Here, the average forwarding probability affected by opinion leaders *p_L_* and the initial susceptible users *S_0_* are all influenced by new susceptible users introduced by opinion leaders, which also confirmed the conclusion before.

**Fig 8:**
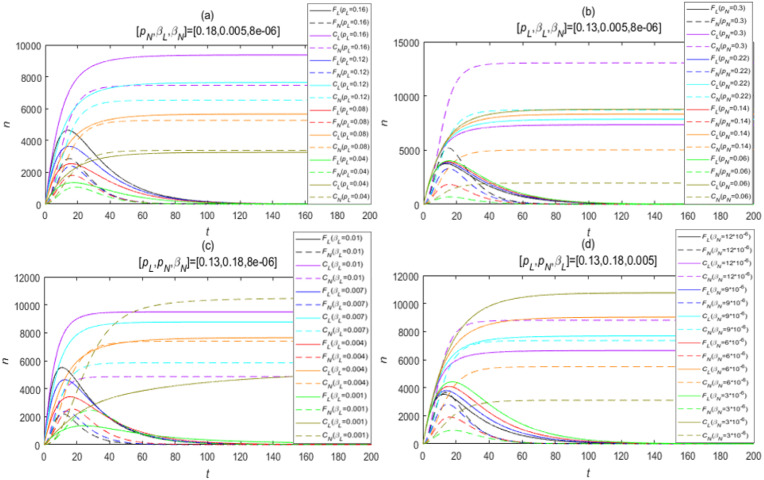
Influence on public opinion index with the change of single parameter. (a) only *p_L_* changes; (b) only *p_N_* changes; (c) only *β_L_* changes; (d) only *β_N_* changes.

Fig 8(a)–8(d) show the excursion span of the value about the four single parameters in each subfigure is illustrated with an equal interval gradient involving the rank from high to low. *p_L_*, *p_N_*, *β_L_*, *β_N_* are four parameters that could best reflect the different influence between opinion leaders and normal users on public opinion. They often play a significant part in determining the coverage of public opinion directly. Therefore, if we want to acquire further conclusion, it is necessary to establish a control group of *F_L_*(*t*), *F_N_*(*t*) as well as *C_L_*(*t*), *C_N_*(*t*) when these four parameters change independently, which is regarded as our further analysis in order to discover the effect on information propagation caused by the crowds with distinct characteristics, and make the different rates more evident between two kinds of population on the public opinion development, as shown in Fig 8(a)–8(d) (the excursion span of the value about the four single parameters in each subfigure is illustrated with an equal interval gradient involving the rank from high to low). According to the overall parameters’ estimation results, we also set the default values of each one considered as *p_L_* = 0.13, *p_N_* = 0.18, *β_L_* = 0.005 and *β_N_* = 8 × 10^−6^. For instance, in Fig 8(a) only *p_L_* changes and the remaining three parameters are all fixed at their default values. The other subfigures are similar to Fig 8(a).

It can be intuitively seen that instantaneous forwarding quantity and the cumulative forwarding quantity show a stronger sensitivity to *β_L_* and *β_N_* compared to the forwarding probability. These two parameters fluctuate only in a very limited range, however, they would cause a drastic change in *F*_*L*_, *F*_*N*_ and *C*_*L*_, *C*_*N*_. So we can draw the following conclusions with an overview of Fig 8:

1. The larger values *p_L_* and *β_L_* have, the higher peak the instantaneous forwarding quantity can reach. With this condition, the final size of the cumulative forwarding population will also be in a stable state in advance; in addition, there is no doubt that the public opinion will break out faster with the increase of *p_L_* and *β_L_*.
2. The forwarding probability *p_L_* of opinion leaders has a positive effect on the trend about instantaneous forwarding quantity *F_L_*(*t*) and cumulative forwarding quantity *C_L_*(*t*); on the contrary, the forwarding probability of normal users *p_N_* shows a slight negative effect on *C_L_*(*t*) and *F_L_*(*t*). This is due to the fact that when *p_N_* rises, a small group of people who originally has a high probability to forward the information of opinion leaders may pull their ship’s head round to forward the information of normal users. In addition, after being influenced by opinion leaders, some users will not forward the information of them, but forward the information of the original post owner, so the cumulative forwarding quantity directly caused by opinion leaders *C_L_*(*t*) will relatively reduce a bit. Definitely, *F_N_*(*t*) and *C_N_*(*t*) are more sensitive to the fluctuation of *p_N_*. We can find out that even though *p_L_* has the half value of *p_N_*, the coverage scale of public opinion caused by the former is approximately equal to the latter or even wider than it, which powerfully proves that opinion leaders have much greater influence to promote the evolvement of public opinion than that produced by normal users.
3. In most of the circumstances, the average exposure rate of opinion leaders *β_L_* also provides a positive effect on these indexes. Owing to the similar principles as the aforementioned content said, the average exposure rate *β_N_* belonging to normal users has a negative effect on *C_L_*(*t*) and *F_L_*(*t*), which is even more severe than that of *p_N_*. From the default floating range of the two types of exposure rate, it is obvious that the chance of an opinion leader being contacted with people is much greater than that of a normal user, and the value of *β_L_* is almost 1000 times than *β_N_*. Part of the reason for this phenomenon stems from the considerable fan base and the strong voice of opinion leaders.

**Fig 9:**
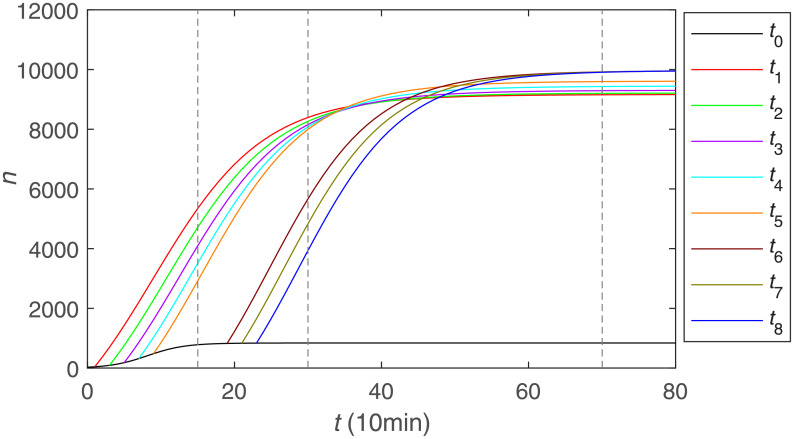
Public opinion dissemination process with opinion leaders join at different times.

Fig 9 shows the public opinion dissemination process with opinion leaders joining at different times, implicating the influence of opinion leaders on the speed of information propagation and the final size of the cumulative forwarding population. It can be seen that during the outbreak of public opinion, the earlier the opinion leaders participate, the greater the cumulative quantity of forwarding will be; during the stable period of public opinion propagation, it will appear the situation similar to the above, and the final size of the cumulative number of forwarding will tend to be larger than that in the circumstance where opinion leaders join in the information propagation during the outbreak. Therefore, if we intend to make one information become popular much faster, we can persuade opinion leaders to get involved in the early stage of the information outbreak as quickly as possible. Moreover, if we mainly hope to broaden the coverage of public opinion and more people contact with the relevant information, we can allow opinion leaders to participate in the stable period of propagation.

## Conclusion

In this paper, we propose an opinion-leader susceptible-forwarding-immune (OL-SFI) dynamics model based on the forwarding quantities generated by opinion leaders and normal users in different stages from the Chinese Sina-microblog during the outbreak of COVID-19. For there are always many opinion leaders with an enormous fan base in a real-world, investigating the propagation mechanism with the influence of opinion leaders is extraordinarily necessary and significant. We take the great driving power of the opinion leaders in the information propagation process into consideration while studying the spreading dynamics of the information within the interference of different numbers of opinion leaders at each active period. The role of our model is to examine how opinion leaders have influenced public opinion and information propagation in different stages when the outbreak unfolds.

We have performed the data fitting based on the real data related to COVID-19 obtained from Chinese Sina-microblog to verify the effectiveness of our model. Based on the results of numerical simulations and sensitivity analysis at different stages, we can use the rules of evolution with regard to characteristic indexes of public opinion to formulate effective interventions for opinion leaders to participate in the public opinion. To a large extent, the average inactive rate *α* in this model is determined by the general public’s character and their interest, so it is more difficult to be controlled. However, we can have an insight into the hidden crisis behind the public opinion by putting average exposure rates and forwarding probabilities into a real-time update strategy. More significantly, the index ℜ*_o_* which is calculated from actual data can be used to derive the thresholds of *p_N_* and *β_N_* utilizing a contrary thinking, so that we could simulate the earlier brewing process of public opinion, and notify the relevant departments to make plans of facing the emergency before the outbreak of public opinion. On the other hand, for some positive information, we are supposed to encourage opinion leaders to forward it several times in order to increase the forwarding probability *p_L_* and average exposure rate *β_L_*. Meanwhile, it can be inferred from the estimation results of cumulative forwarding quantity that the best time and the appropriate number of opinion leaders for the intervention in different stages. Consequently, we are able to use the “Golden Stage” to maximize the positive influence of those opinion leaders. As for some bad information, we need to reduce the exposure rate of normal users *β_N_* avoiding more people to read such information, and at the same time *α_N_* could be increased. In this way, it is more conducive to creating a good direction for the development of public opinion and avoiding sharpening conflicts between different kinds of people. Furthermore, it is vital to pay attention to *C*_∞_ by doing real-time detection of its tendency combined with the results of PRCC analysis. If we perceive that some pieces of harmful information have entered the situation where the public opinion is well prepared to break out, *β_N_* and *p_N_* should be declined as soon as possible. Simultaneously, it is necessary to stop the participation of opinion leaders by preventing *p_L_* increasing successively to make the number of susceptible users *S_0_* decrease, so as to avoid the harmful public opinion to become a more severe deterioration.

In a word, the propagation scale can be gradually promoted with the participation of opinion leaders, and their entering time will control the forwarding lifeline. That is, the earlier opinion leaders get into the intervention, the greater their influence will be. Our focus here is to make use of our OL-SFI dynamics model to help original post owners design effective communication strategies for the on-going battle against COVID-19 and future pandemic outbreaks in terms of public opinion and information dissemination.
